# Supplementary material for: Validation of a battery of inhibitory control tasks reveals a multifaceted structure in non-human primates
Source: PeerJ. 2022 Feb 9;10:e12863. doi: 10.7717/peerj.12863 (PMC8840138; doi:10.7717/peerj.12863)
Supplement: Supplemental Information 1 — Confounding factors were divided in individual (sex, age, rank and experience with picture) and experimental determinants (session and time point). All full models included the individual ID as a random factor. The Estimates (representing the change in the dependent variable relative to the baseline category of each predictor variable), Standard Error, t-value and p-value using maximum likelihood method. The variables in bold stimulus, age, trial and time point had a significant effect on the models. 4,094 data points were analysed. Note. Number of subjects 21 Likelihood-ratio test comparing the best fitted model (with session, time point and age as explanatory variables) with the null model : χ2 4 = 296.02, p < 0.0001. The subjects had a longer response latency as they get older (χ2 1 = 9.086, p < 0.01), and their response latency were shorter as session (χ2 1 = 4.798, p < 0.05) and time point (χ2 4 = 276.165, p < 0.0001) increased. [file peerj-10-12863-s001.docx]

***log(response latency) on a trial***

| **Predictor** | **Estimate** | **Std. Error** | **t-value** | **p-value** |
| --- | --- | --- | --- | --- |
| (Intercept) | 8.223 | 0.213 | 38.526 | 0.000 |
| Stimulus picture | 0.112 | 0.035 | 3.189 | **0.001** |
| Picture Object vs face | 0.018 | 0.025 | 0.753 | 0.452 |
| Picture Threat vs neutral | -0.058 | 0.043 | -1.350 | 0.177 |
| Sex male | 0.120 | 0.150 | 0.801 | 0.436 |
| Age | 0.043 | 0.018 | 2.296 | **0.035** |
| Rank low vs high | 0.196 | 0.154 | 1.273 | 0.220 |
| Experience  picture | -0.084 | 0.097 | -0.862 | 0.388 |
| Trial | 0.000 | 0.002 | 0.069 | 0.944 |
| Session | -0.035 | 0.017 | -2.20 | **0.027** |
| Time point | -0.534 | 0.032 | -16.877 | **0.000** |
