# Supplementary material for: Validation of a battery of inhibitory control tasks reveals a multifaceted structure in non-human primates
Source: PeerJ. 2022 Feb 9;10:e12863. doi: 10.7717/peerj.12863 (PMC8840138; doi:10.7717/peerj.12863)
Supplement: Supplemental Information 2 — Confounding factors were divided in individual (sex, age and rank) and experimental determinants (session and time point). All full models included the individual ID as a random factor. The Estimates (representing the change in the dependent variable relative to the baseline category of each predictor variable), Standard Error, z-value and p-value using maximum likelihood method. The type of stimulus (Go or No-go) and session had a significant effect on the models. 7,783 data points were analysed. Note. Number of subjects 20 Likelihood-ratio test comparing the best fitted model (with type of stimulus and session as explanatory variables) with the null model: χ2 2 = 3335.6, p < 0.0001. The success on a trial was higher as the number of the session increased: χ2 1 = 6.172, p < 0.05. [file peerj-10-12863-s002.docx]

***Success on a trial***

| **Predictor** | **Estimate** | **Std. Error** | **t-value** | **p-value** |
| --- | --- | --- | --- | --- |
| (Intercept) | 6.824 | 0.671 | 11.072 | 0.000 |
| Stimulus No-Go | -7.098 | 0.413 | -17.196 | **0.000** |
| Sex female | -0.300 | 0.200 | -1.5000 | 0.134 |
| Age | -0.004 | 0.037 | -0.111 | 0.911 |
| Rank low vs high | 0.023 | 0.364 | 0.065 | 0.948 |
| Trial | 0.001 | 0.004 | 0.173 | 0.862 |
| Session | 0.081 | 0.033 | 2,456 | **0.014** |
| Time point | 0.091 | 0.093 | 0.972 | 0.331 |
