# Supplementary material for: Validation of a battery of inhibitory control tasks reveals a multifaceted structure in non-human primates
Source: PeerJ. 2022 Feb 9;10:e12863. doi: 10.7717/peerj.12863 (PMC8840138; doi:10.7717/peerj.12863)
Supplement: Supplemental Information 3 — Confounding factors were divided in individual (sex, age and rank) and experimental determinants (session and time point). All full models included the individual ID as a random factor. The Estimates (representing the change in the dependent variable relative to the baseline category of each predictor variable), Standard Error, t-value and p-value using maximum likelihood method. Only the variable in bold stimulus had a significant effect on the models. 7,783 data points were analysed. Note. Number of subjects 20 Likelihood-ratio test comparing the best fitted model (with type of stimulus as explanatory variables) with the null model: χ2 1 = 600.73, p < 0.001. [file peerj-10-12863-s003.docx]

***log(response latency) on a trial***

| **Perdictor** | **Estimate** | **Std. Error** | **z-value** | **p-value** |
| --- | --- | --- | --- | --- |
| (Intercept) | 7.638 | 0.160 | 47.800 | 0.000 |
| Stimulus  No-Go | 0.509 | 0.020 | 24.969 | **0.000** |
| Sex female | -0.041 | 0.042 | -0.967 | 0.334 |
| Age | 0.006 | 0.012 | 0.555 | 0.579 |
| Rank low vs high | 0.115 | 0.116 | 0.999 | 0.331 |
| Trial | -0.001 | 0.001 | -0.969 | 0.332 |
| Session | 0.010 | 0.006 | 1.640 | 0.101 |
| Time point | 0.001 | 0.018 | 0.068 | 0.946 |
