# Supplementary material for: Validation of a battery of inhibitory control tasks reveals a multifaceted structure in non-human primates
Source: PeerJ. 2022 Feb 9;10:e12863. doi: 10.7717/peerj.12863 (PMC8840138; doi:10.7717/peerj.12863)
Supplement: Supplemental Information 4 — Confounding factors were divided in individual (sex, age and rank) and experimental determinants (session and time point). All full models included the individual ID as a random factor. The Estimates (representing the change in the dependent variable relative to the baseline category of each predictor variable), Standard Error, z-value and p-value using maximum likelihood method. The variables in bold rule, trial and session had a significant effect on the models. 6,686 data points were analysed. Note. Number of subjects 19 Likelihood-ratio test comparing the best fitted model with the null model: χ2 4 = 27.74, p < 0.001. The success on a trial was higher as the trials (χ2 1 = 4.101, p < 0.05) and session increased ( χ2 1 = 11.687, p < 0.05 ). [file peerj-10-12863-s004.docx]

**Success on a trial**

| **Predictor** | | **Estimate** | | **Std. Error** | | **z value** | | **p-value** | |  | |
| --- | --- | --- | --- | --- | --- | --- | --- | --- | --- | --- | --- |
| (Intercept) | | 0.797 | | 0.272 | | 2.885 | | 0.004 | |  | |
| Ruverse rule | | -0.099 | | 0.069 | | -1.437 | | 0.150 | |  | |
| Sex female | -0.185 | | 0.157 | | -1.183 | | 0.237 | |  | |  |
| Age | | -0.025 | | 0.016 | | -1.607 | | 0.108 | |  | |
| Rank low vs high | | -0.025 | | 0.149 | | -0.170 | | 0.897 | |  | |
| Trial | | 0.005 | | 0.002 | | 2.047 | | **0.041** | |  | |
| Session | | 0.056 | | 0.017 | | 3.377 | | **0.001** | |  | |
| Time point | | -0.088 | | 0.072 | | -1.233 | | 0.218 | |  | |
