# Supplementary material for: Validation of a battery of inhibitory control tasks reveals a multifaceted structure in non-human primates
Source: PeerJ. 2022 Feb 9;10:e12863. doi: 10.7717/peerj.12863 (PMC8840138; doi:10.7717/peerj.12863)
Supplement: Supplemental Information 5 — Confounding factors were divided in individual (sex, age, rank and experience with picture) and experimental determinants (session and time point). All full models included the individual ID as a random factor. The Estimates (representing the change in the dependent variable relative to the baseline category of each predictor variable), Standard Error, t-value and p-value using maximum likelihood method. Only the variables in bold sex, session and time point had a significant effect on the models. 346 data points were analysed. [file peerj-10-12863-s005.docx]

***Distraction control score***

| **Predictor** | **Estimate** | **Std. Error** | **t-value** | **p-value** |
| --- | --- | --- | --- | --- |
| (Intercept) | -185.419 | 683.239 | -0.271 | 0.786 |
| Sex male | -1368.504 | 631.269 | -2.172 | **0.045** |
| Age | -102.284 | 103.125 | -0.986 | 0.334 |
| Rank low vs high | -1211.880 | 619.867 | -1.955 | 0.068 |
| Experience with pictures | 317.939 | 856.463 | 0.371 | 0.715 |
| Trial | 6.374 | 9.807 | 0.649 | 0.517 |
| Session | 455.514 | 124.249 | 3.666 | **0.0003** |
| Time point | 2019.521 | 205.8753 | 9.815 | **0.000** |
| Type picture Object | -117.94 | 245.685 | -0.484 | 0.628 |
| Type picture Threat | 66.953 | 249.686 | 0.266 | 0.790 |
