# Supplementary material for: Validation of a battery of inhibitory control tasks reveals a multifaceted structure in non-human primates
Source: PeerJ. 2022 Feb 9;10:e12863. doi: 10.7717/peerj.12863 (PMC8840138; doi:10.7717/peerj.12863)
Supplement: Supplemental Information 6 — Confounding factors were divided in individual (sex, age and rank) and experimental determinants (session and time point). All full models included the individual ID as a random factor. The Estimates (representing the change in the dependent variable relative to the baseline category of each predictor variable), Standard Error, t-value and p-value using maximum likelihood method. None of the variables had a significant effect on the models. 120 data points were analysed. [file peerj-10-12863-s006.docx]

***Action control score***

| **Predictor** | **Estimate** | **Std. Error** | **t-value** | **p-value** |
| --- | --- | --- | --- | --- |
| (Intercept) | 47.742 | 13.653 | 3.497 | 0.001 |
| Sex male | -10.487 | 9.604 | -1.092 | 0.292 |
| Age | 0.744 | 1.061 | 0.701 | 0.494 |
| Rank low vs high | -3.680 | 9.857 | -0.373 | 0.714 |
| Session | 4.631 | 2.851 | 1.646 | 0.103 |
| Time point | 3.062 | 4.370 | 0.631 | 0.523 |
