# Supplementary material for: Validation of a battery of inhibitory control tasks reveals a multifaceted structure in non-human primates
Source: PeerJ. 2022 Feb 9;10:e12863. doi: 10.7717/peerj.12863 (PMC8840138; doi:10.7717/peerj.12863)
Supplement: Supplemental Information 7 — Confounding factors were divided in individual (sex, age, rank) and experimental determinants (session and time point). The Estimates (representing the change in the dependent variable relative to the baseline category of each predictor variable), z-value and p-value using maximum likelihood method. None of the variables had a significant effect on the model. 38 data points were analysed. [file peerj-10-12863-s007.docx]

***Rule control score***

| **Predictor** | **Estimate** | **Std. Error** | **t-value** | **p-value** |
| --- | --- | --- | --- | --- |
| (Intercept) | 40.211 | 68.579 | 0.586 | 0.566 |
| Sex male | -109.509 | 65.719 | -1.666 | 0.118 |
| Age | 6.471 | 8.011 | 0.808 | 0.4323 |
| Rank low vs high | -18.676 | 72.299 | -0.258 | 0.799 |
| Time point | -2.001 | 13.721 | -0.146 | 0.887 |
