# Supplementary material for: Validation of a battery of inhibitory control tasks reveals a multifaceted structure in non-human primates
Source: PeerJ. 2022 Feb 9;10:e12863. doi: 10.7717/peerj.12863 (PMC8840138; doi:10.7717/peerj.12863)
Supplement: Supplemental Information 8 — Distraction control score (Distraction task), Action control score (Go/No-go) and Rule control score (Reversal Learning) are represented. ✔ indicates that the individual’s performances are significantly repeatable between tasks. *p < 0.05, **p < 0.01, ***p < 0.001 [file peerj-10-12863-s008.docx]

| Contextual R  for the scores | **Distraction**  **control** | **Action control** | **Rule control** |
| --- | --- | --- | --- |
| **Distraction control** | 1 | - | - |
| **Action control** | - 0.166   (p = 2.69e-05)*** | 1 | - |
| **Rule control** | - 0.212   (p = 9.46e-04) *** | - 0.138   (p = 0.012)* | 1 |
