# Supplementary material for: Validation of a battery of inhibitory control tasks reveals a multifaceted structure in non-human primates
Source: PeerJ. 2022 Feb 9;10:e12863. doi: 10.7717/peerj.12863 (PMC8840138; doi:10.7717/peerj.12863)
Supplement: Supplemental Information 9 — Confounding factors were divided in individual (sex, age and rank) and experimental determinants (session and time point). The Estimates (representing the change in the dependent variable relative to the baseline category of each predictor variable t-value and p-value using maximum likelihood method. The variables in bold session and time point had a significant effect on the models. 237 data points were analysed. [file peerj-10-12863-s009.docx]

| **Predictor** | **Estimate** |  | **Std. Error** | **t-value** | **p-value** |
| --- | --- | --- | --- | --- | --- |
| (Intercept) | -0.241 |  | 0.226 | -1.065 | 0.282 |
| Task Go/No-go | 0.012 |  | 0.096 | 0.128 | 0.898 |
| Sex male | -0.381 |  | 0.162 | -2.338 | 0.032 |
| Age | 0.007 |  | 0.020 | 0.320 | 0.752 |
| Rank low vs high | 0.051 |  | 0.156 | 0.330 | 0.741 |
| Session | 0.126 |  | 0.058 | 2.160 | **0.032** |
| Time point | 0.268 |  | 0.096 | 2.809 | **0.006** |
