# Supplementary material for: Validation of a battery of inhibitory control tasks reveals a multifaceted structure in non-human primates
Source: PeerJ. 2022 Feb 9;10:e12863. doi: 10.7717/peerj.12863 (PMC8840138; doi:10.7717/peerj.12863)
Supplement: Supplemental Information 10 — Confounding factors were divided in individual (sex, age and rank) and experimental determinants (session and time point). All full models included the individual ID as a random factor. The Estimates (representing the change in the dependent variable relative to the baseline category of each predictor variable t-value and p-value using maximum likelihood method. Only the variable in bold sex had a significant effect on the model (when comparing with the full model). 158 data points were analysed. [file peerj-10-12863-s010.docx]

| **Predictor** | **Estimate** | **Std.Error** | **t-value** | **p-value** |
| --- | --- | --- | --- | --- |
| (Intercept) | -0.325 | 0.367 | -0.890 | 0.375 |
| Task Reversal learning | 0.183 | 0.219 | 0.838 | 0.404 |
| Sex male | -0.528 | 0.218 | -2.494 | **0.023** |
| Age | 0.030 | 0.025 | 1.230 | 0.221 |
| Rank low vs high | -0.108 | 0.226 | -0.447 | 0.656 |
| Session | 0.163 | 0.112 | 1.457 | 0.148 |
| Time point | 0.122 | 0.162 | 0.751 | 0.454 |
