# Supplementary material for: Validation of a battery of inhibitory control tasks reveals a multifaceted structure in non-human primates
Source: PeerJ. 2022 Feb 9;10:e12863. doi: 10.7717/peerj.12863 (PMC8840138; doi:10.7717/peerj.12863)
Supplement: Supplemental Information 11 — Confounding factors were divided in individual (sex, age and rank) and experimental determinants (session and time point). All full models included the individual ID as a random factor. The Estimates (representing the change in the dependent variable relative to the baseline category of each predictor variable), Standard Error, t-value and p-value using maximum likelihood method. The variable in bold had a significant effect on the models. 155 data points were analysed. [file peerj-10-12863-s011.docx]

| **Predictor** | **Estimate** | **Std. Error** | **t-value** | **p-value** |
| --- | --- | --- | --- | --- |
| (Intercept) | -0.325 | 0.365 | -0.890 | 0.375 |
| Task Reversal learning | 0.173 | 0.217 | 0.792 | 0.425 |
| Sex male | -0.416 | 0.133 | -3.140 | **0.023** |
| Age | 0.006 | 0.016 | 0.396 | 0.206 |
| Rank low vs high | -0.108 | 0.224 | -0.478 | 0.633 |
| Session | 0.166 | 0.11 | 1.430 | 0.128 |
| Time point | 0.1209 | 0.165 | 0.759 | 0.448 |
